# Supplementary material for: Transcriptional regulators ensuring specific gene expression and decision-making at high TGFβ doses
Source: Life Sci Alliance. 2024 Nov 14;8(1):e202402859. doi: 10.26508/lsa.202402859 (PMC11565188; doi:10.26508/lsa.202402859)
Supplement: Supplementary file 1 [file LSA-2024-02859_TableS1.docx]

**Table S1: Absolute number of EMT and non-EMT genes in DDGs and non-DDGs,** *related to main Figure 2,* The EMT gene list was curated, and the following target genes related to EMT were added: *WNT9A* (Gasior *et al.*, 2017; Zhang *et al.*, 2018), *AMIGO2* (Kanda *et al.*, 2017; Tanio *et al.*, 2021), *COL4A1* (Miyake *et al.*, 2017; Cui, Shan and Qiao, 2022; Tian *et al.*, 2023), *EPCAM* (Hyun *et al.*, 2016; Sankpal *et al.*, 2017), *FAP* (Wu *et al.*, 2020; Ping *et al.*, 2023), *LAMB3* (Liu *et al.*, 2019; Zhang *et al.*, 2019), *LAMA3* (Huang and Chen, 2021; Islam *et al.*, 2023), *PLAU* (Chen *et al.*, 2021; Wu *et al.*, 2022), *ITGB6* (Thomas, Nyström and Marshall, 2006; Zheng *et al.*, 2021), *SULF2* (Vicente *et al.*, 2015; Tao *et al.*, 2017), *TEAD2* (Diepenbruck *et al.*, 2014), *LTBP2* (Wan *et al.*, 2017; Wang *et al.*, 2018).

|  | **EMT genes** | **Non-EMT** |
| --- | --- | --- |
| **DDGs** (86) | 29 | 57 |
| **Non-DDGs** (4737) | 142 | 4595 |
| ***total*** | *171* | *4652* |
