# Supplementary material for: Transcriptional regulators ensuring specific gene expression and decision-making at high TGFβ doses
Source: Life Sci Alliance. 2024 Nov 14;8(1):e202402859. doi: 10.26508/lsa.202402859 (PMC11565188; doi:10.26508/lsa.202402859)
Supplement: Supplementary file 2 [file LSA-2024-02859_TableS2.docx]

Table S2. GSEA of DDGs considering KEGG (Kyoto Encyclopedia of Genes and Genomes) and REAC (Reactome) gene sets with adjusted p-value< 0.01, *related to main Figure 2, see supplementary method: GSEA of DDGs*

| **Source and term name** | **Adjusted p-value** | **Number of DDGs intersecting with gene set** | **Gene symbols** |
| --- | --- | --- | --- |
| **KEGG**, ECM-receptor interaction | 1.1886E-05 | 7 | *COL4A1, FN1, ITGB6, LAMA3, LAMB3, LAMC2, TNC* |
| **KEGG**, HPV infection | 1.6827E-05 | 11 | *COL4A1, FN1, FZD2, ITGB6, LAMA3, LAMB3, LAMC2, LFNG, PIK3CD, TNC, WNT9A* |
| **KEGG**, Focal adhesion | 0.00027557 | 8 | *COL4A1, FN1, ITGB6, LAMA3, LAMB3, LAMC2, PIK3CD, TNC* |
| **KEGG**, Small cell lung cancer | 0.00030181 | 6 | *COL4A1, FN1, LAMA3, LAMB3, LAMC2, PIK3CD* |
| **KEGG**, Amoebiasis | 0.00051999 | 6 | *COL4A1, FN1, LAMA3, LAMB3, LAMC2, PIK3CD* |
| **KEGG**, PI3K-Akt-Signaling pathway | 0.01610993 | 8 | *COL4A1, FN1, ITGB6, LAMA3, LAMB3, LAMC2, PIK3CD ,TNC* |
| **REAC**, Non-integrin membrane-ECM interactions | 0.00040216 | 6 | *COL4A1, FN1, LAMA3, LAMB3, LAMC2, TNC* |
| **REAC**, Anchoring fibril formation | 0.00056117 | 4 | *COL4A1, LAMA3, LAMB3, LAMC2* |
| **REAC**, ECM proteoglycans | 0.00185292 | 6 | *COL4A1, FN1, ITGB6, LAMA3, SERPINE1, TNC* |
| **REAC**, Extracellular matrix organization | 0.00301222 | 10 | *COL4A1, FN1, ITGB6, LAMA3, LAMB3, LAMC2, LTBP2, LTBP3, SERPINE1, TNC* |
| **REAC**, Signaling by TGF-beta Receptor Complex | 0.00535432 | 6 | *ITGB6, LTBP2, LTBP3, PMEPA1, SERPINE1, SKIL* |
| **REAC**, MET promotes cell motility | 0.00336972 | 4 | *FN1, LAMA3, LAMB3, LAMC2* |
