# Supplementary material for: Transcriptional regulators ensuring specific gene expression and decision-making at high TGFβ doses
Source: Life Sci Alliance. 2024 Nov 14;8(1):e202402859. doi: 10.26508/lsa.202402859 (PMC11565188; doi:10.26508/lsa.202402859)
Supplement: Supplementary file 3 [file LSA-2024-02859_TableS3.docx]

Table S3. Number of differentially regulated genes with phenotypic association, *see Methods part: Bioinformatics analysis of bulk RNA sequencing data described or rejected by the simple model, related to main Figure 3*

|  | all genes | EMT-related | cell cycle-related |
| --- | --- | --- | --- |
| differentially regulated by TGFβ | 4823 | 172 | 391 |
| described by activation model | 919 (19 %) | 41 (24 %) | 52  (13 %) |
| described by inhibition model | 2160 (45 %) | 37 (22 %) | 183  (46 %) |
| rejected | 1744 (36 %) | 93 (54 %) | 156  (39 %) |
