# Supplementary material for: Transcriptional regulators ensuring specific gene expression and decision-making at high TGFβ doses
Source: Life Sci Alliance. 2024 Nov 14;8(1):e202402859. doi: 10.26508/lsa.202402859 (PMC11565188; doi:10.26508/lsa.202402859)
Supplement: Supplementary file 4 [file LSA-2024-02859_TableS4.docx]

**Table S4.** Filter settings defining numbers of genes belonging to dynamic gene groups, *related to main Figure 4*

For a gene to belong to a group its RNA sequencing time course had to fulfill the indicated filter settings upon high dose (100 pM) TGFβ stimulation. For instance, to classify a gene with delayed kinetics its absolute (abs.) fold change (FC) needed to be greater than 2.5 in at least one of the time points 180 or 360 or 720 or 1440 min post-stimulation. Simultaneously, the absolute fold change for early time points (45 min and 90 min) needed to be smaller than 1.5. For a gene to belong to the inconsistent gene group its absolute fold change needed to higher than 1.5 and simultaneously lower than 0.8 in at least one of the time points (45/90/180/360/720/1440 min).

| kinetics | Selected filter settings | Number of genes rejected  *(1744)* | Number of genes described by inhibition model  *(2160)* | Number of genes described by activation model *(919)* |
| --- | --- | --- | --- | --- |
| I) Delayed | *180 min OR 360 min OR 720 min OR 1440 min abs. FC >2.5, 45 min AND 90 min abs. FC < 1.5* | 483 ↓189 ↑294 | 277 | 131 |
| II) Inconsistent | *45 OR 90 OR 180 OR 360 OR 720 OR 1440 min FC > 1.5*  *AND*  *45 OR 90 OR 180 OR 360 OR 720 OR 1440 min FC < 0.8* | 315 | 3 | 6 |
| III) Biphasic | *45 OR 90 min abs. FC >1.5,  abs. FC 180 min < 90 min, 180 min abs. FC < 1.5,  360 OR 720 OR 1440 min abs. FC >1.5*  *180 min abs. FC >1.5,  abs. FC 360 min < 180 min, 360 min abs. FC < 0.58,  720 OR 1440 min abs. FC > 1.5* | 112 ↓48 ↑64 | 16 | 22 |
| IV) Continuous | *90 min AND 180 min AND 360 min AND 720 min AND 1440 min abs. FC >1.5* | 66 ↓8 ↑58 | 1 | 26 |
| V) Immediate early | *45 min OR 90 min abs. FC >1.5, 180 min AND 360 min OR 720 min AND 1440 min abs. FC < 1.5* | 45  ↓25 ↑20 | 1 | 5 |
