# Supplementary material for: Transcriptional regulators ensuring specific gene expression and decision-making at high TGFβ doses
Source: Life Sci Alliance. 2024 Nov 14;8(1):e202402859. doi: 10.26508/lsa.202402859 (PMC11565188; doi:10.26508/lsa.202402859)
Supplement: Supplementary file 5 [file LSA-2024-02859_TableS5.docx]

**Table S5.** Absolute number and percentages of genes classified in different dynamical gene groups described by the extended model versions model 1-8. There are rejected genes grouped in more than one gene group. In total, 1577 out of 1744 target genes are described by the extended model versions, *related to main Figure 4, D, E, see Methods part: Feed-Forward loops equation, FFL model fitting, FFL model fitting selection*

|  | **Model 1** | **Model 2** | **Model 3** | **Model**  **4** | **Model 5** | **Model 6** | **Model 7** | **Model 8** | **Total**  **(%)** |
| --- | --- | --- | --- | --- | --- | --- | --- | --- | --- |
| **Biphasic** (absolute)  (%) | 1  0.9 | 19  17.0 | 20  17.9 | 9  8 | 5  4.5 | 5  4.5 | 14  12.5 | 1  0.9 | 74  66.2 |
| **Continuous**  (absolute)  (%) | 4  6.1 | 18  27.3 | 4  6.1 | 17  25.8 | 4  6.1 | 3  4.5 | 2  3 | 2  3 | 54  81.9 |
| **Delayed**  (absolute)  (%) | 39  8.1 | 42  8.7 | 99  20.5 | 102  21.1 | 2  0.4 | 6  1.2 | 79  16.4 | 66  13.7 | 435  89.8 |
| **Inconsistent**  (absolute)  (%) | 0  0 | 42  13.3 | 107  34 | 102  32.4 | 0  0 | 3  1 | 17  5.4 | 0  0 | 271  86.1 |
| **IEG**  (absolute)  (%) | 0  0 | 6  13.3 | 11  24.4 | 5  11.1 | 0  0 | 7  15.6 | 1  2.2 | 0  0 | 30  65.2 |
| **DDGs**  (absolute)  (%) | 6  10.9 | 4  7.3 | 6  10.9 | 17  30.9 | 1  1.8 | 2  3.6 | 6  10.9 | 4  7.3 | 46  83.6 |
| **ungrouped**  (absolute)  (%) | 10  1.2 | 72  8.4 | 226  26.4 | 198  23.2 | 14  1.6 | 66  7.7 | 168  19.6 | 53  6.2 | 807  94.4 |
| **Total**  (absolute)  (%) | 54  3.4 | 183  11.6 | 428  27.1 | 409  25.9 | 25  1.6 | 88  5.6 | 268  17 | 122  7.7 | **1577**  **90.4** |
