# Supplementary material for: Transcriptional regulators ensuring specific gene expression and decision-making at high TGFβ doses
Source: Life Sci Alliance. 2024 Nov 14;8(1):e202402859. doi: 10.26508/lsa.202402859 (PMC11565188; doi:10.26508/lsa.202402859)
Supplement: Supplementary file 6 [file LSA-2024-02859_TableS6.docx]

Table S6. Quantified slopes and Pearson correlation coefficient (R) of scatter plots (gene expression control vs. gene expression KD) for each time point, *related to main Figure 5, see Methods part: Assessing impact of co-factor KD on target gene expression (slope quantification)*

| **Factor KD** | **Time point post-stimulation (min)** | **Slope control vs. KD (DESeq2)** | | **Slope  control vs. KD (DESeq2)** | **Variance confidence interval** | **Pearson Correlation coefficient (R), ctrl vs. KD** |
| --- | --- | --- | --- | --- | --- | --- |
| **SMAD2** | 90 | 0.75 | 0.74 | | [0.74, 1.32] | 0.94 |
|  | 180 | 0.80 | 0.80 | | [0.60 1.60] | 0.95 |
|  | 360 | 0.64 | 0.64 | | [0.97 1.02] | 0.93 |
|  | 720 | 0.63 | 0.61 | | [0.90, 1.11] | 0.92 |
| **SMAD3** | 90 | 0.77 | 0.74 | | [0.87, 1.14] | 0.94 |
|  | 180 | 0.86 | 0.82 | | [0.60 1.56] | 0.96 |
|  | 360 | 0.72 | 0.73 | | [0.98 1.02] | 0.94 |
|  | 720 | 0.81 | 0.76 | | [0.88, 1.13] | 0.92 |
| **SNAI1** | 90 | 1.29 | 1.10 | | [0.93, 1.07] | 0.87 |
|  | 180 | 0.99 | 0.93 | | [0.92 1.08] | 0.88 |
|  | 360 | 1.03 | 0.89 | | [0.96 1.05] | 0.88 |
|  | 720 | 1.06 | 1.03 | | [0.88, 1.12] | 0.93 |
| **SNAI2** | 90 | 1.23 | 1.17 | | [0.88, 1.13] | 0.88 |
|  | 180 | 1.15 | 1.10 | | [0.87 1.14] | 0.90 |
|  | 360 | 0.99 | 1.01 | | [0.92 1.09] | 0.92 |
|  | 720 | 1.06 | 1.02 | | [0.94, 1.06] | 0.93 |
| **RUNX1** | 90 | 1.16 | 1.13 | | [0.94, 1.06] | 0.95 |
|  | 180 | 1.14 | 1.00 | | [0.91 1.10] | 0.94 |
|  | 360 | 1.46 | 1.39 | | [0.77 1.28] | 0.87 |
|  | 720 | 1.06 | 1.03 | | [0.93, 1.07] | 0.96 |
| **SKIL** | 90 | 1.26 | 1.21 | | [0.86, 1.16] | 0.95 |
|  | 180 | 1.01 | 0.96 | | [0.93 1.07] | 0.97 |
|  | 360 | 0.94 | 0.94 | | [0.99 1.00] | 0.95 |
|  | 720 | 0.95 | 0.92 | | [0.87, 1.14] | 0.95 |
| **SKI** | 90 | 1.60 | 1.58 | | [0.41, 1.96] | 0.92 |
|  | 180 | 1.09 | 1.00 | | [0.85 1.17] | 0.93 |
|  | 360 | 1.23 | 1.28 | | [0.71 1.4] | 0.91 |
|  | 720 | 1.10 | 1.04 | | [0.97, 1.03] | 0.97 |
| **JUNB** | 90 | 1.03 | 1.03 | | [0.89, 1.12] | 0.94 |
|  | 180 | 0.91 | 0.84 | | [0.84 1.18] | 0.89 |
|  | 360 | 0.90 | 0.93 | | [0.91 1.09] | 0.94 |
|  | 720 | 0.74 | 0.73 | | [0.78, 1.26] | 0.93 |
| **JUN** | 90 | 0.94 | 0.94 | | [0.75, 1.30] | 0.96 |
|  | 720 | 1.02 | 0.97 | | [0.91, 1.10] | 0.96 |
| **KLF10** | 90 | 0.96 | 0.95 | | [0.81, 1.22] | 0.96 |
|  | 720 | 0.90 | 0.88 | | [0.78, 1.26] | 0.98 |
| **ATF3** | 90 | 1.07 | 1.06 | | [0.79, 1.24] | 0.97 |
|  | 720 | 0.96 | 0.95 | | [0.79, 1.24] | 0.99 |
