# Supplementary material for: Transcriptional regulators ensuring specific gene expression and decision-making at high TGFβ doses
Source: Life Sci Alliance. 2024 Nov 14;8(1):e202402859. doi: 10.26508/lsa.202402859 (PMC11565188; doi:10.26508/lsa.202402859)
Supplement: Supplementary file 7 [file LSA-2024-02859_TableS7.docx]

Table S7. Number of cells analyzed for SMAD2 nuc/cyt ratio in control, non-targeting-control and KD MCF10A cells (SKI, SKIL, and JUNB KD), *related to supplementary Figure S6, C, see supplementary method: SMAD2 live-cell imaging upon TGFβ stimulated transcription factor KD cells*

| **Condition** | **replicate** | **Cell number** |
| --- | --- | --- |
| **WT, unstimulated** | R1 | 792 |
|  | R2 | 465 |
|  | R3 | 562 |
| **NTC, unstimulated** | R1 | 589 |
|  | R2 | 446 |
|  | R3 | 679 |
| **SKI, unstimulated** | R1 | 386 |
|  | R2 | 152 |
|  | R3 | 203 |
| **JUNB, unstimulated** | R1 | 539 |
|  | R2 | 162 |
|  | R3 | 257 |
| **SKIL, unstimulated** | R1 | 206 |
|  | R2 | 383 |
|  | R3 | 418 |
| **WT, stimulated** | R1 | 642 |
|  | R2 | 373 |
|  | R3 | 468 |
| **NTC, stimulated** | R1 | 524 |
|  | R2 | 339 |
|  | R3 | 428 |
| **SKI, stimulated** | R1 | 227 |
|  | R2 | 216 |
|  | R3 | 205 |
| **JUNB, stimulated** | R1 | 438 |
|  | R2 | 243 |
|  | R3 | 282 |
| **SKIL, stimulated** | R1 | 562 |
|  | R2 | 304 |
|  | R3 | 378 |
