# Supplementary material for: Transcriptional regulators ensuring specific gene expression and decision-making at high TGFβ doses
Source: Life Sci Alliance. 2024 Nov 14;8(1):e202402859. doi: 10.26508/lsa.202402859 (PMC11565188; doi:10.26508/lsa.202402859)
Supplement: Supplementary file 8 [file LSA-2024-02859_TableS8.docx]

**Table S8.** JUNB binding sites in SERPINE1 and FN1 promoter region

| **target gene** | **start location** | **end location** | **binding site** | **strand** |
| --- | --- | --- | --- | --- |
| FN1 | 1110 | 1122 | TAATGATTCAAGC | - |
| FN1 | 1652 | 1664 | GCCTGACACATTA | + |
| FN1 | 1110 | 1122 | GCTTGAATCATTA | + |
| SERPINE1 | 91 | 103 | CGGTGGCTCATGC | + |
| SERPINE1 | 889 | 901 | GCTTGAATCATCC | + |
| SERPINE1 | 1843 | 1855 | ACATGCCTCAGCA | + |
| SERPINE1 | 889 | 901 | GGATGATTCAAGC | - |
